# A Fermented Milk Product Containing *B. lactis* CNCM I-2494 Improves the Tolerance of a Plant-Based Diet in Patients with Disorders of Gut–Brain Interactions

**DOI:** 10.3390/nu13124542

**Published:** 2021-12-18

**Authors:** Boris Le Nevé, Adrian Martinez-De la Torre, Julien Tap, Adoración Nieto Ruiz, Muriel Derrien, Aurélie Cotillard, Jean-Michel Faurie, Elizabeth Barba, Marianela Mego, Quentin Dornic, John Butler, Xavi Merino, Beatriz Lobo, Ferran Pinsach Batet, Marta Pozuelo, Javier Santos, Francisco Guarner, Chaysavanh Manichanh, Fernando Azpiroz

**Affiliations:** 1 Danone Nutricia Research, 91767 Palaiseau, France; Julien.TAP@danone.com (J.T.); Muriel.DERRIEN@danone.com (M.D.); aurelie.cotillard@danone.com (A.C.); Jean-Michel.FAURIE@danone.com (J.-M.F.); quentin.dornic@orange.fr (Q.D.); 2 Digestive System Research Unit, University Hospital Vall d’Hebron, 08035 Barcelona, Spain; adriadelatorre@hotmail.com (A.M.-D.l.T.); anieto@vhebron.net (A.N.R.); ebarbaorozco@gmail.com (E.B.); marianelamego@hotmail.com (M.M.); xavier.merino@uab.cat (X.M.); beatriz.lobo@vhir.org (B.L.); f.pinsach.batet@gmail.com (F.P.B.); mpozud00@gmail.com (M.P.); javier.santos@vhir.org (J.S.); fguarner@icloud.com (F.G.); cmanicha@gmail.com (C.M.); 3 Centro de Investigación Biomédica en Red de Enfermedades Hepáticas y Digestivas (Ciberehd), Departament de Medicina, Universitat Autònoma de Barcelona, 08193 Cerdanyola del Vallés, Spain; 4 Departament de Medicina, Universitat Autònoma de Barcelona, 08193 Cerdanyola del Vallès, Spain; 5 Department of Gastroenterology, Hospital Clínic, 08036 Barcelona, Spain; 6 Lawson Health Research Institute, London ON N6C 2R5, Canada; jbutler@lawsonimaging.ca

**Keywords:** flatulence, fermentable carbohydrates, probiotics, microbiota, digestive symptoms, disorders of gut–brain interactions, *B. lactis* DN-173010

## Abstract

Healthy, plant-based diets, rich in fermentable residues, may induce gas-related symptoms. The aim of this exploratory study was to assess the effects of a fermented milk product, containing probiotics, on the tolerance of a healthy diet in patients with disorders of gut–brain interactions (DGBI), complaining of excessive flatulence. In an open design, a 3-day healthy, mostly plant-based diet was administered to patients with DGBI (52 included, 43 completed) before and at the end of 28 days of consumption of a fermented milk product (FMP) containing *Bifidobacterium animalis* subsp. *lactis* CNCM I-2494 and lactic acid bacteria. As compared to a habitual diet, the flatulogenic diet increased the perception of digestive symptoms (flatulence score 7.1 ± 1.6 vs. 5.8 ± 1.9; *p* < 0.05) and the daily number of anal gas evacuations (22.4 ± 12.5 vs. 16.5 ± 10.2; *p* < 0.0001). FMP consumption reduced the flatulence sensation score (by –1.6 ± 2.2; *p* < 0.05) and the daily number of anal gas evacuations (by –5.3 ± 8.2; *p* < 0.0001). FMP consumption did not significantly alter the overall gut microbiota composition, but some changes in the microbiota correlated with the observed clinical improvement. The consumption of a product containing *B. lactis* CNCM I-2494 improved the tolerance of a healthy diet in patients with DGBI, and this effect may be mediated, in part, by the metabolic activity of the microbiota.

## 1. Introduction

A healthy diet requires a proper balance of nutrients, vitamins and minerals for one’s own metabolism, but also many residues that serve as substrates for the intestinal microbiota, the largest pool of symbiotic microorganisms in the human body. The metabolic processing of diet residues by the microbiota involves fermentative pathways that release gas, and in susceptible individuals, this may be associated with the perception of digestive symptoms.

A large proportion of patients in clinical practice complain of digestive symptoms, such as bloating, abdominal distension, and flatulence, in the absence of structural abnormalities, and attribute their complaints to intestinal gas. These patients exhibit susceptibility of the alimentary tract, with poor tolerance of residue-rich healthy diets. This may have further implications because some data indicate that these patients have enteric dysbiosis, and a diet poor in residues may further deteriorate their microbiota.

Previous studies indicate that the administration of specific living microorganisms (probiotics) may improve gas-related symptoms. Indeed, it has been shown that a fermented milk product containing *Bifidobacterium animalis* subsp. *lactis* CNCM I-2494 and lactic acid bacteria improves symptoms and well-being in women with mild digestive complaints [1,2,3,4], and reduces bloating, digestive discomfort, and abdominal distension in IBS-C patients [5,6]. Furthermore, the mechanism of action of this probiotic seems to be related to the metabolism of the microbiota [7].

The aim of the present study was to determine the efficacy of a fermented milk product (FMP) containing *B. lactis* CNCM I-2494 and lactic acid bacteria on the tolerance of a healthy diet in susceptible individuals. To this aim, we recruited a pool of patients with moderate to severe symptoms in response to a healthy diet containing an adequate load of plant residues, and evaluated the effect of the FMP on subjective clinical parameters (gas-related symptoms) and objective physiological parameters related to intestinal gas metabolism.

## 2. Materials and Methods

### 2.1. Study Design and General Procedure

**Figure 1:**
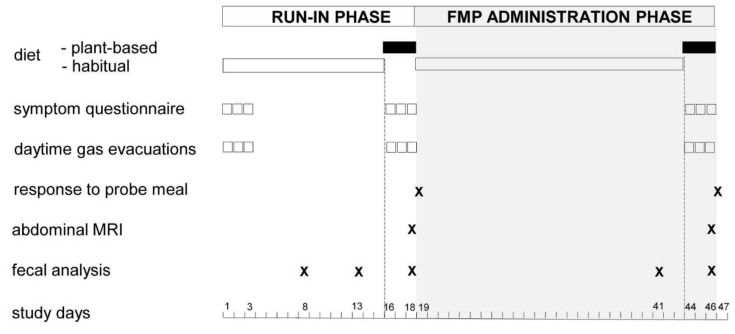
Study design. FMP: fermented milk product. Abdominal MRI scheduled in half of the participants.

Single-centre, open-label study performed in a tertiary care referral centre on the effect of a fermented milk product (FMP) on intestinal gas production and gastrointestinal symptoms. The study consisted of an 18-day run-in phase and a 28-day FMP administration phase (Figure 1). The main outcomes were gastrointestinal symptoms, number of anal gas evacuations, and volume of anal gas evacuated following a probe meal measured at the end of the run-in phase and the administration phase.

During the study, participants were instructed to consume their habitual diet except during the last 3 days of the run-in phase (days 16–18) and the administration phase (days 44–46) when a mostly plant-based diet (see below) was administered. Participants were not allowed to consume any fermented dairy products (with or without probiotics) or any tablets, pills, or food supplements containing pre- or probiotics other than those provided during the study. The food items of the plant-based diet and the fermented milk product were provided by the investigators.

The clinical study was conducted according to the Declaration of Helsinki. The study protocol had previously been approved by the Institutional Review Board of the University Hospital Vall d’Hebron (Comitè d’Ètica d’Investigació Clinica, Vall d’Hebron Insititut de Recerca; protocol number PR-AG 292/2012, approved 29 January 2016) and all participants provided written informed consent. The protocol was also registered with ClinicalTrials.gov (NCT02936713) and included separate pilot studies in healthy subjects [8] and in patients with disorders of gut–brain interactions (reported here). All authors had access to the study data and reviewed and approved the final manuscript.

### 2.2. Participants

Patients with DGBI (both genders; 18–75 year age range; body mass index (BMI) 18.5–30 kg/m^2^) diagnosed by Rome III criteria participated in the study using previously validated clinical questionnaires [9,10,11,12]. Patients were recruited from the outpatient clinics, both on campus and in the community, serviced by the Vall d’Hebron Digestive Diseases Department. Before inclusion, patients were required to have flatulence (≥5 on a 0–10 scale), abdominal bloating or discomfort (≥3 on a 0–10 scale) and negative sensation of digestive well-being during the previous week. The day after the run-in phase (day 19) the symptom questionnaires related to days 16–18 (on the plant-based diet) were reviewed, and the following continuation criteria were required to enter the administration phase: (a) prospective confirmation of entry criteria (described above), (b) ≥8 anal gas evacuations per day, and (c) ≥50% daily compliance to the plant-based diet (calculated as the percent intake per day of the total fibre content in the diet). Intake of antibiotics during the previous two months, changes in dietary habits in the previous 4 weeks, antecedents of digestive surgery (except for appendectomy and cholecystectomy performed more than two years before), and treatments that might affect the central nervous system or gastrointestinal function (anxiolytics/antidepressants and laxatives at stable dose were allowed) were considered as exclusion criteria.

### 2.3. Plant-Based Diet

The plant-based diet consisted of the following: (a) breakfast of wholemeal cookies (39 g) plus coffee, tea and/or milk; (b) lunch of white beans (200 g), lentils (200 g) or chickpeas (200 g) plus meat, fowl, fish or eggs, and fruit (banana, figs, peaches, or prunes); (c) dinner of vegetable cream (200 mL) plus meat, fowl or fish, and fruit (apple or pear). This diet provides 58% caloric content as carbohydrates, 29% as proteins and 13% as fat with 19 g of fibre per day. Caloric content of the diet was not standardized. Participants were instructed to self-report the foods they consumed during the 3 days on the plant-based diet to assess compliance.

### 2.4. Study Product

During the 28-day administration phase, participants consumed 1 pot (125 g) of the study product at breakfast and another pot at dinner. The study product was a fermented milk containing three *Streptococcus salivarius* subsp. *thermophilus* strains (CNCM I-2773, CNCM I-2130, CNCM I-2272), *Lactobacillus delbrueckii* subsp. *bulgaricus* (CNCM I-1519), *Bifidobacterium animalis* subsp. *lactis* (CNCM I-2494 previously referenced as DN-173010), and *Lactococcus lactis* subsp. *lactis* (CNCM I-1631). The study product was manufactured and supplied by Danone Nutricia Research, Palaiseau, France and contained per g at least 3.4 × 10^7^ colony forming units (cfu) of *B. lactis*, 1 × 10^6^ cfu of *L*. *lactis*, and 1 × 10^7^ cfu of *S. thermophilus* and *L. bulgaricus*.

### 2.5. Main Outcomes

Gastrointestinal symptoms and the number of anal gas evacuations were measured during 3-day periods at the following 3 time points throughout the study: (a) at the beginning of the run-in phase on the habitual diet (days 1–3); (b) at the end of the run-in phase on the plant-based diet (days 16–18); (c) at the end of the administration phase on the plant-based diet (days 44–46) (Figure 1). The volume of anal gas evacuated after a probe meal was measured after 3 days on the plant-based diet at the following 2 time points: (a) the day after the run-in phase (day 19); (b) the day after the administration phase (day 47).

#### 2.5.1. Daily Symptoms Questionnaire

During the 3 days of each evaluation period, the following gastrointestinal symptoms were self-assessed by the participants using daily questionnaires (0–10 analogue scoring scales): (a) subjective sensation of flatulence; (b) abdominal bloating; (c) abdominal distension; (d) borborygmi; (e) odoriferous flatus; (f) abdominal discomfort/pain. The questionnaire also recorded the following: (g) digestive well-being scored on a scale graded from +5 (extremely pleasant sensation/satisfaction) to −5 (extremely unpleasant sensation/dissatisfaction); (h) frequency of bowel movements; (i) stool consistency using the Bristol stool form scale. The sensitivity and quality of this patient-related outcome have been validated by previous research aiming to assess the effects of dietary interventions on both healthy subjects and patients with DGBIs [12].

#### 2.5.2. Number of Anal Gas Evacuations

The number of daytime anal gas evacuations was assessed during each evaluation period. Participants were instructed to carry an event marker (Hand Tally Counter No 101, Digi Sport Instruments, Shangqiu, China) and to use it to record each passage of anal gas during the day. Previous research showed that this method provides reproducible results [12,13] that correlate with the simultaneous recording of anal gas outflow (R > 0.95; *p* < 0.05) [14].

#### 2.5.3. Volume of Anal Gas Production after a Probe Meal (Gas Production Test)

Participants came to the laboratory the morning after an overnight fast to consume a probe meal consisting of whole meal cookies (96 g) plus coffee and milk (54 g carbohydrates, 7 g proteins and 21 g fat with 8 g fibre; 450 Kcal). The volume of anal gas evacuated was measured continuously during the 4 h after the probe meal, as previously described [12,13]. Briefly, anal gas was collected using a rectal balloon catheter (20 F Foley catheter, Bard, Barcelona, Spain) connected via a gas-tight line to a barostat, and the volume was continuously measured. To prevent anal gas leaks, the intrarectal balloon was inflated with 5 mL of water.

### 2.6. Exploratory Outcomes

#### 2.6.1. Colonic Gas Content

Half of the participants were scheduled for colonic gas content measurements. Colonic gas content during consumption of the plant-based diet was measured by abdominal magnetic resonance imaging (MRI) in the run-in phase (day 18) and in the administration phase (day 46), following the procedure described previously [8].

#### 2.6.2. Faecal Microbiota Analysis

All participants were given a kit with standard instructions to self-collect their stool samples at home at the following 5 time points throughout the study: (a) during run-in phase on the habitual diet (2 samples on days 8 and 13); (b) during run-in phase on the plant-based diet (day 18); (c) during administration phase on the habitual diet (day 41); (d) during administration phase on the plant-based diet (day 46) (Figure 1). Stool samples were mixed with a spatula provided in the kit to obtain a homogenous mixture, immediately frozen by the participants in their home freezers at −20 °C and later brought to the laboratory in a freezer pack, where they were stored at −80 °C until further use. Genomic DNA was extracted by mechanical process [15] and faecal microbiota were profiled using 16S rRNA gene amplicon sequencing based on Illumina MiSeq technology. Amplicon reads were analysed using QIIME software (1.9.1). Sequences were clustered based on the USEARCH (search and clustering) algorithm (5.2.236v) into operational taxonomic units (OTUs), taxonomically assigned using a database combining greengenes (gg_13_8 release) and PATRIC (Pathosystems Resource Integration Center).

### 2.7. Statistical Analysis

Due to the exploratory nature of this study, no sample size calculation or multiplicity adjustments were performed. Analysed populations for the run-in and administration phases were the full analysis set 1 (FAS 1; *n* = 47) and FAS 2 (*n* = 43), respectively.

Clinical results are expressed as mean [95% CI]. For each parameter, the values of the 2 last days from each evaluation period were averaged for statistical comparisons. Comparisons were performed using Wilcoxon signed-rank test. Statistical tests were two sided with a significance level of 5% and all confidence intervals are presented as two sided with a confidence level of 95%.

Microbial ecology and statistical analyses were performed using QIIME and R software (3.4.3v). Spearman correlations were used to assess correlations between changes in clinical parameters and faecal microbiota composition, as previously described [8].

## 3. Results

### 3.1. Demographics and Compliance to Study Procedures

**Figure 2:**
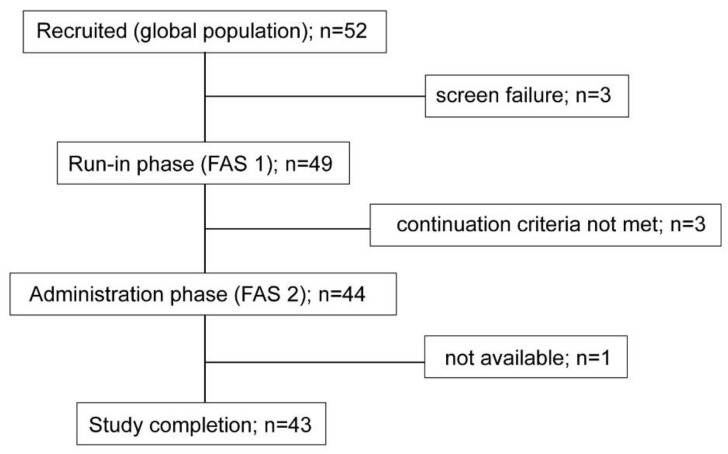
Flow-chart. FAS 1: full analysis set 1; FAS 2: full analysis set 2.

Forty-nine subjects with DGBI (39 women, 10 men; 51 ± 15 years; 24.9 ± 3.5 Kg/m^2^ BMI) were included in the run-in phase (FAS 1); 38 patients fulfilled the criteria of irritable bowel syndrome (20 constipation-predominant, 8 diarrhoea-predominant, 7 mixed type, 3 undetermined), 5 had functional abdominal pain, 3 had functional bloating, and 3 had functional dyspepsia (postprandial distress syndrome). After the run-in phase, 44 patients fulfilled the continuation criteria and entered the administration phase (FAS 2), and 43 completed the study (Figure 2). Adherence to the dietary instructions was high (mean per-protocol compliance >90%) and the study product was well tolerated.

### 3.2. Effect of the Plant-Based Diet during the Run-In Phase

On their habitual diet, patients reported digestive symptoms (flatulence, abdominal discomfort, abdominal distension, bloating, and borborygmi) and odoriferous flatus (Table 1). These symptoms were associated with a negative sensation of digestive well-being. On the plant-based diet, digestive symptoms significantly increased with a decrease in the sensation of digestive well-being (Table 1). These subjective changes were associated with a significant increase in the number of daytime anal gas evacuations, without changes in stool frequency and consistency (Table 1).

### 3.3. Effect of the Fermented Milk Product on the Tolerance of the Plant-Based Diet

**Figure 3:**
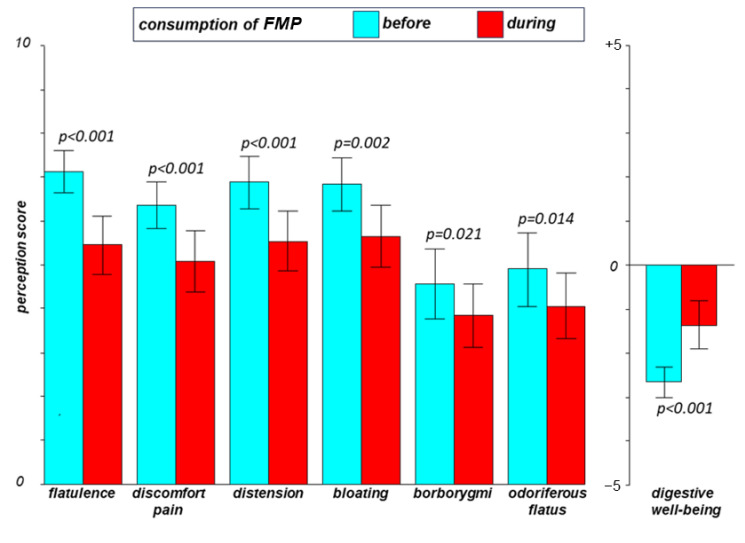
Effect of consumption of fermented milk product (FMP) on tolerance of a plant-based diet. Data are means [95% confidence interval]. Statistical comparisons were performed by Wilcoxon signed-rank test in each subject using the average of the last 2 days of evaluation periods (on the plant-based diet) before (days 17 and 18) and during FMP consumption (days 45 and 46: *n* = 43).

Consumption of the study product improved the tolerance of the healthy plant-based diet, with a significant decrease in digestive symptoms and enhanced sensation of digestive well-being (Figure 3). This effect was associated with a reduction in the number of anal gas evacuations (by −5.3 [−7.8; −2.7] evacuations per day; *p* < 0.001).

No effects of FMP consumption were detected on stool frequency and consistency (changes by −0.08 [−0.2; 0.0] daily bowel movements and 0.17 [−0.2; 0.5] Bristol score), the volume of gas collected during the 4 h period after ingestion of the probe meal (139 mL [109; 169] before versus 153 mL [77; 229] during administration; change by 13 mL [−65; 91]), and the volume of colonic gas measured by MRI (total volume of 208 mL [157; 258] before versus 191 mL [125; 256] during administration; change by −16.65 mL [−77; 43]; paired data from 22 participants).

### 3.4. Faecal Microbiota

A total of 199 faecal samples collected from the participants were used for the microbial community analysis. No differences in the overall gut microbiota composition among the five faecal samples taken during the study were detected by the weighted UniFrac distance. FMP consumption did not elicit significant changes in the faecal microbiota diversity, as shown by the alpha- and beta-diversity (Wilcoxon signed-rank tests, *p* > 0.05 for Chao1 index, *p* > 0.05 for weighted UniFrac distance).

**Figure 4:**
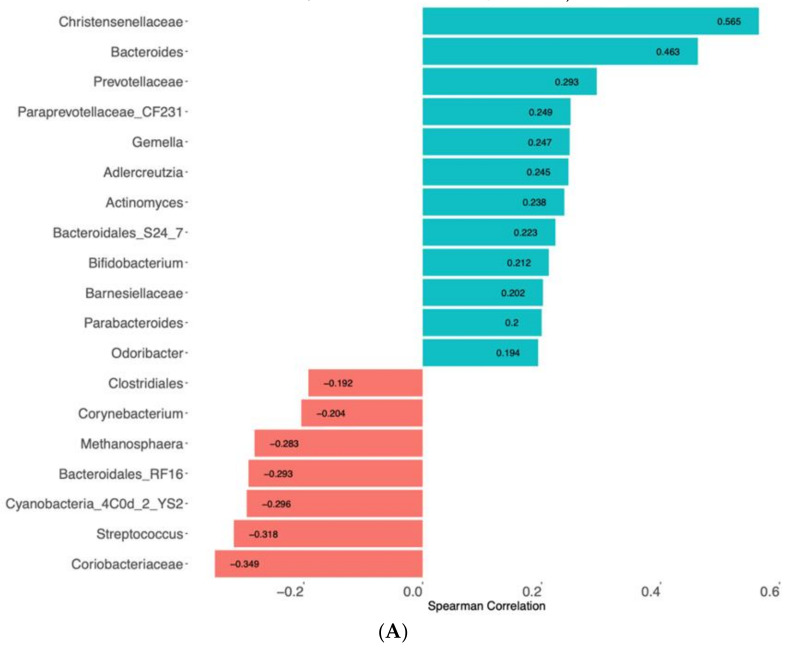

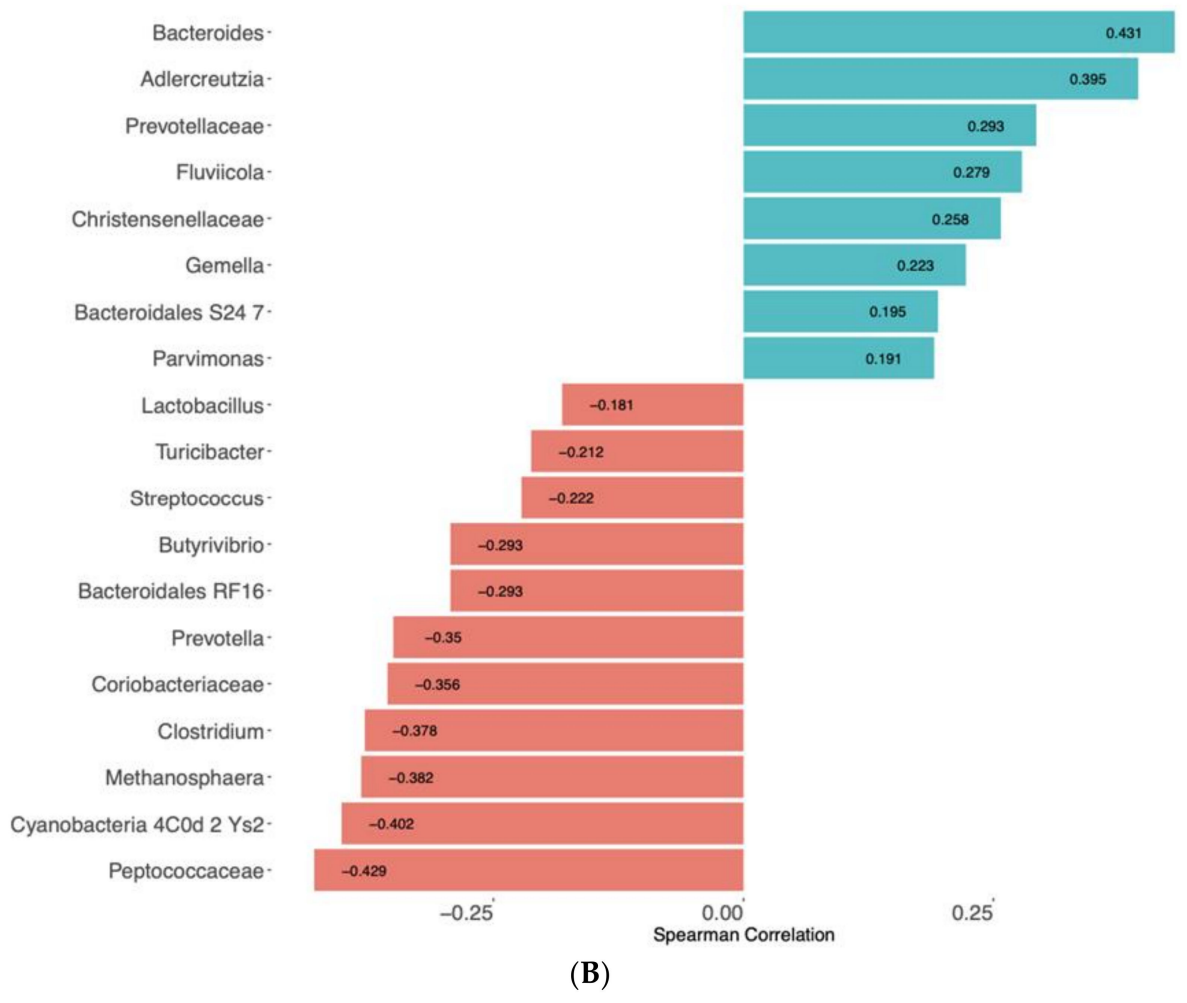
Spearman correlation between changes in clinical parameters and bacterial genera. (**A**). Number of anal gas evacuations. (**B**). Sensation of flatulence. Strength and significance of Spearman correlations (rho coefficient) are indicated in the colour shade. Red: change in microbiota genera negatively associated with the change in clinical parameter. Blue: change in microbiota genera positively associated with the change in clinical parameter. Only the highest correlations are shown.

Spearman correlation was used to associate the response of the clinical parameters to FMP consumption with changes in faecal microbiota composition. The decrease in the number of anal gas evacuations during FMP consumption correlated with a decrease in the relative abundance of *Christensenellaceae* (rho = 0.56; *p* < 0.001) and *Bacteroides* (rho = 0.46; *p* < 0.006), and an increase in *Coriobacteriaceae* (rho = −0.34; *p* = 0.042) (Figure 4A). The decrease in flatulence sensation was related to a decrease in the relative abundance of *Bacteroides* spp. (rho = 0.43; *p* = 0.010) and *Adlercreutzia* (rho = 0.39; *p* = 0.020), and an increase in *Peptococcaceae* (rho = −0.43; *p* = 0.011), *Cyanobacteria 4C0d 2 Ys2* (rho = −0.40; *p* = 0.018), and *Methanosphaera* (rho = −0.28; *p* = 0.025) (Figure 4B).

## 4. Discussion

Our data indicate that the consumption of a fermented milk product (FMP), containing *B. lactis* CNCM I-2494 and lactic acid bacteria, improves symptoms in patients with disorders of gut–brain interactions (DGBI) on a healthy, plant-based diet. Digestive sensations depend on the balance between the gut content (intraluminal stimuli) and tolerance, i.e., the way the intraluminal content is sensed and managed by the gut. Conceivably, the product improved the tolerance of a healthy plant-based diet acting on these two factors.

When consuming the plant-based diet, patients experienced worsening of their symptoms, and this may be related to the load of fibre, fermentable oligo-, di-, mono-saccharides and polyols (FODMAPs), and other non-absorbable residues. These residues are metabolized by the microbiota, releasing gas, and increase the bulk of colonic biomass, and both mechanisms may be related to the poor tolerance of the diet. Indeed, on the plant-based diet, the number of anal gas evacuations increased, but FMP consumption corrected this effect, indicating that the FMP reduced the amount of gas released from the fermentation of dietary residues. By contrast, in the gas production test, the volume of gas collected during the 4 h after the probe meal was not influenced by the FMP. This apparent discrepancy may be explained by the overflow of fermentable residues delivered with the probe meal, which might have overcome the potential effect of the product on fermentative activity. The volume of gas within the colon, measured by magnetic resonance, remained constant before and during FMP consumption. This observation, i.e., the effect of the FMP on gas metabolism, without changes in the colonic gas content, is in accordance with previous studies that showed no differences in intracolonic gas volume with dietary interventions that modified gas production [16,17]. Indeed, the gas content is under tight homeostatic control at around 100–200 mL. The amount of gas released by fermentation varies greatly depending on the availability of residues. However, the gas production is matched with a very dynamic and effective gas disposal system. The most important mechanism is absorption across the gut–blood barrier and elimination by pulmonary diffusion via breath. About 70% of the gas produced is rapidly eliminated following this pathway. The hydrogen concentration in the breath was not measured in the present study, but a product with the same *B. lactis* CNCM I-2494 reduced hydrogen breath excretion after a nutrient meal containing lactulose was given to IBS patients [7]. A fraction of gas is eliminated from the lumen by gas-consuming microorganisms; lactate-producing bacteria present in the FMP may contribute to microbiota gas consumption, but this metabolic process conceivably takes longer, and may not be appreciated shortly after the probe meal.

It has been documented that, as compared to a low-residue diet, a high-residue diet increases both the volume of colonic biomass and faecal clearance, conceivably by an increase in the residue load and microbiota proliferation [16,17]. FMP administration may have moderated this effect, but biomass volumes and faecal weight were not measured in the present study, and we cannot determine whether the effect of the FMP on symptoms was related to the downregulation of biomass proliferation.

In addition to the potential intraluminal effects of the FMP, probiotics may have influenced the way the intestine handled and sensed its content, i.e., intestinal motility and sensitivity. Patients with functional gut disorders exhibit visceral hypersensitivity and reflex dysfunctions; through the interaction of these mechanisms, they develop symptoms in response to stimuli, e.g., diets that are well tolerated by healthy subjects. It has been shown that the administration of a product with the same *B. lactis* CNCM I-2494 reduced visceral sensitivity to colorectal distension in an experimental rat model [18], and exerted a modulatory effect on the brain regions involved in the central processing of emotion and sensation in healthy women [19]. Furthermore, administration of the same product to patients with IBS-C had a prokinetic effect (accelerated orocecal and colonic transit), associated with an improvement in symptoms [5].

Our 4-week FMP intervention had minor effects on faecal microbiota composition. In a previous study, using shotgun metagenomics, we observed that strong dietary interventions for short periods of time have only minor effects on composition, but a considerable impact on microbiota metabolism, as assessed by changes in the microbial metabolic pathways in faecal samples, and the urinary excretion of microbial metabolites [16]. Our findings in this study may also reflect metabolic adaptation to the plant-based diet during FMP consumption, as shown by the reduction in flatulence scores and anal gas evacuations after FMP consumption, linked with the downregulation of some putative hydrogen-producing taxa (*Bacteroides, Christensenellaceae,* and *Prevotellaceae*) and the upregulation of some hydrogen-consuming taxa (*Methanospherae*). In a previous study, an FMP, containing *B. lactis* CNCM I-2494, reduced the metabolic potential of the *Prevotella/Bacteroides* ratio in IBS patients [7]. Here, we could detect a reduction in *Prevotellaceae,* associated with improved clinical effects, particularly in relation to the number of anal gas evacuations and the sensation of flatulence. A high-fermented-food diet was recently shown to increase faecal microbiota diversity in individuals consuming a median of five fermented food servings per day (including FMPs) for 10 weeks [20]. Our study did not reproduce such findings, most likely due to the shorter duration and lower diversity and/or exposure of the fermented food in our intervention.

### Limitations 

We acknowledge that this pilot study was not controlled, and the results must be considered as hypothesis-generating, rather than conclusive. A possible placebo effect on subjective perception cannot be excluded; however, the patients did not know what to expect in response to the consumption of the plant-based diet and the product. Several technical limitations, discussed above, had to be compromised due to the complexity of the study and the relatively large sample size. In particular, the microbiota analysis by 16S rRNA gene sequencing does not allow for the evaluation of the potential (and plausible) effects of the FMP on microbiota metabolic pathways. Furthermore, we did not evaluate the effect of the product at different times during and after administration, and, hence, we cannot ascertain whether the product may induce sensations by itself that could confound the effects observed on the plant-based diet, in terms of how long it should be administered to be effective and whether the effects are persistent after discontinuation.

## 5. Clinical Inferences and Conclusions

The current management of gas-related symptoms in patients with disorders of gut–brain interactions includes low-residue diets and prebiotics [21,22,23,24,25,26]. The present study shows that probiotics may offer an alternative approach. Conceivably, the treatment of these patients may benefit from an individualized combination of diet, prebiotics, and probiotics, based on objective indicators related to physiological parameters and microbiota activity.

## Figures and Tables

**Table 1:** Effects of the plant-based diet on gas-related symptoms and frequency of anal gas evacuations.

	Habitual Diet (*n* = 48)	Plant-Based Diet (*n* = 47)	*p* Value
Flatulence *	5.8 [5.2; 6.32]	6.8 [6.2; 7.4]	*p* < 0.001
Abdominal discomfort/pain *	5.3 [4.7; 5.9]	6.2 [5.7; 6.7]	*p* < 0.001
Abdominal distension *	5.8 [5.1; 6.4]	6.7 [6.1; 7.3]	*p* < 0.001
Bloating *	5.7 [5.0; 6.3]	6.7 [6.1; 7.3]	*p* < 0.001
Borborygmi *	4.0 [3.2; 4.8]	4.5 [3.8; 5.3]	*p* < 0.001
Odor of flatus *	4.3 [3.5; 5.1]	4.7 [3.9; 5.5]	*p* >0.05
Digestive well-being *	−1.8 [−2.3; −1.2]	−2.5 [−2.9; −2.1]	*p* < 0.005
Anal gas evacuations *	16.5 [13.5; 19.4]	22.3 [18.6; 26.0]	*p* < 0.001
Bowel movements **	1.4 [1.2; 1.6]	1.4 [1.2; 1.6]	*p* > 0.05
Stool consistency **	4.0 [3.6; 4.3]	4.0 [3.6; 4.4]	*p* > 0.05

Data are means [95% confidence interval] presented on full analysis set 1 (FAS1) population; comparisons by Wilcoxon signed-rank test. Data calculated in each subject using average over last 2 days of each evaluation period (*) or average over last 3 days of each evaluation period (**).

## Data Availability

Data were uploaded in NCBI with the following accession number: PRJNA789678.
